# Immunization with Radiation-Attenuated *Plasmodium berghei* Sporozoites Induces Liver cCD8α^+^DC that Activate CD8^+^T Cells against Liver-Stage Malaria

**DOI:** 10.1371/journal.pone.0005075

**Published:** 2009-04-04

**Authors:** Ousman Jobe, Gina Donofrio, Guangping Sun, Dmitry Liepinsh, Robert Schwenk, Urszula Krzych

**Affiliations:** Department of Cellular Immunology, Division of Malaria Vaccine Development, Walter Reed Army Institute of Research, Silver Spring, Maryland, United States of America; Initiative for Vaccine Research, Switzerland

## Abstract

Immunization with radiation (γ)-attenuated *Plasmodia* sporozoites (γ-spz) confers sterile and long-lasting immunity against malaria liver-stage infection. In the *P. berghei* γ-spz model, protection is linked to liver CD8^+^ T cells that express an effector/memory (T_EM_) phenotype, (CD44^hi^CD45RB^lo^CD62L^lo^ ), and produce IFN-γ. However, neither the antigen presenting cells (APC) that activate these CD8^+^ T_EM_ cells nor the site of their induction have been fully investigated. Because conventional (c)CD8α^+^ DC (a subset of CD11c^+^ DC) are considered the major inducers of CD8^+^ T cells, in this study we focused primarily on cCD8α^+^ DC from livers of mice immunized with *Pb* γ-spz and asked whether the cCD8α^+^ DC might be involved in the activation of CD8^+^ T_EM_ cells. We demonstrate that multiple exposures of mice to *Pb* γ-spz lead to a progressive and nearly concurrent accumulation in the liver but not the spleen of both the CD11c^+^NK1.1^−^ DC and CD8^+^ T_EM_ cells. Upon adoptive transfer, liver CD11c^+^NK1.1^−^ DC from *Pb* γ-spz-immunized mice induced protective immunity against sporozoite challenge. Moreover, in an *in vitro* system, liver cCD8α^+^ DC induced naïve CD8^+^ T cells to express the CD8^+^ T_EM_ phenotype and to secrete IFN-γ. The in vitro induction of functional CD8^+^ T_EM_ cells by cCD8α^+^ DC was inhibited by anti-MHC class I and anti-IL-12 mAbs. These data suggest that liver cCD8α^+^ DC present liver-stage antigens to activate CD8^+^ T_EM_ cells, the pre-eminent effectors against pre-erythrocytic malaria. These results provide important implications towards a design of anti-malaria vaccines.

## Introduction

Dendritic cells (DC) are key conductors in the orchestration of the immune responses to a variety of pathogens [1]. A number of DC subsets have been identified, including conventional (c)CD8α^+^DC, cCD8α^−^DC and plasmacytoid (p)DC and these appear to perform unique functional activities. For example, cCD8α^+^ DC efficiently cross present cell-associated Ags particularly to CD8^+^ T cells [2], [3]; cCD8α^−^ DC usually promote the activation of CD4^+^ T cells [4]; and pDC tend to respond to viral infections [5] and may be involved in the activation of Treg cells [6]. In addition, the distribution of the DC subsets is unique for lymphoid and non-lymphoid organs [7], [8] and this can lead to an organ-specific response often dictated by the tropism of infectious agents. Owing to their prominent role in directing the immune system, DC have been shown to be indispensable for the induction of protective immune responses to a variety of infections [9] , including malaria that is caused by Plasmodia ssp. infected mosquitoes.

Prime-boost immunizations with radiation-attenuated (γ) Plasmodia sporozoites (γ-spz) constitutes the most effective method for inducing long-lived, sterile protection against the pre-erythrocytic stages (sporozoite and liver) of both rodent and human malaria [10], [11]. We have reported that *Plasmodium berghei* (*Pb*) γ-spz-induced protection against liver-stage infection is mediated primarily by intrahepatic IFN-γ-producing effector/memory CD44^hi^CD45RB^lo^CD62L^lo^ CD8^+^T cells (CD8^+^T_EM_) [12]. However, the mechanisms of activation of intrahepatic CD8^+^T_EM_ cells require further investigation. For example, the identity of the APC that process and present liver-stage Ags for the induction of the CD8^+^T_EM_ cells remains to be elucidated. Several studies conducted in the past have revealed a critical role for DC in the induction of anti-Plasmodia protection against pre-erythrocytic stage Ags. It has been demonstrated that DC-depleted mice fail to develop protection following γ-spz immunization [13]. Bone-marrow derived dendritic cells pulsed with *Pb* γ-spz or *Pb*-derived antigen induced antigen-specific CD8^+^ T cell responses [14]. Similary, adoptive-transfer of DC loaded with *Plasmodium yoelii* (*Py*) circumsporozoite (CS) protein peptides into BALB/c mice results in the generation of peptide-specific CD8^+^ T cells and reduction of parasitemia following infectious challenge [15]. More recently, it has been shown that DC prime *Py* CS protein specific TCR Tg CD8^+^ T cells in the LN draining the site of the mosquito bite, however, the CD8^+^ T cell effector function following infectious sporozoite challenge occurs in the liver and is DC independent [16]. These and other studies comprise a significant body of work on the roles of splenic [17], [18] and bone marrow-derived DC [19], [20] but comparable studies of liver DC have not been carried out, in part because of the paucity of these cells. Nonetheless, liver DC have been shown to play an important role in liver immunity [21] but their involvement in the induction of protective immunity to the liver stage of malaria remains unknown and, hence, requires investigation. In addition, the relative contributions of the various liver DC subsets also remain to be elucidated. Like infectious spz, *Pb* γ-spz infect hepatocytes but the parasites arrest at the liver-stage of development, which leads to the apoptosis of the infected hepatocytes [22]. In contrast, infectious sporozoites do not cause infected hepatocytes to undergo apoptosis and are ineffective in inducing protective CD8^+^ T cells [23], [24]. Because numerous studies [2], [3], [25], [26] have shown that cCD8α^+^ DC in particular can ingest exogenous cell-associated Ag and cross-present it to CD8^+^ T cells, it is reasonable to assume, therefore, that γ-spz-induced apoptotic hepatocytes are internalized by hepatic DC [22] and the available liver-stage Ags are cross-presented to hepatic CD8^+^ T cells. We asked therefore whether the cCD8α^+^ subset of liver DC is involved in the induction of the effector CD8^+^ T cells that mediate protection against *Pb* sporozoite challenge.

Herein, we present evidence that cCD8α^+^DC accumulate concurrently with CD8^+^ T_EM_ cells in the livers of *Pb* γ-spz-immunized mice. We also show that these cells are able to activate naïve CD8^+^T cells to express the T_EM_ phenotype (CD44^hi^CD45RB^lo^CD62L^lo^). Therefore, we propose that cCD8α^+^DC are involved in the induction of the liver-stage antigen-specific CD8^+^ T cells, the key effectors against liver-stage malaria.

## Results

### The numbers of CD11c^+^NK1.1^−^ DC are low in the livers of naïve mice, but increase following prime-boost immunizations with *Pb* γ−spz

Liver CD8^+^ T cells are the key effectors in protection induced by *Pb* γ*−* spz [27]; however, neither the cellular/molecular processes leading to the induction of these cells nor the sites where this induction occurs have been thoroughly investigated. For example, it is not fully understood whether DC and/or other APC are involved in the presentation of liver-stage Ags for the activation of CD8^+^ T cells. Because γ-spz enter hepatocytes, where they undergo an aborted development, it is reasonable to assume that Ags responsible for the induction of effector CD8^+^ T cells are expressed primarily in the liver [28]. Because DC are considered the most efficient professional APC, we presumed, therefore, that liver DC might be excellent APC candidates for presentation of liver-stage Ags to CD8^+^ T cells.

**Figure 1:**
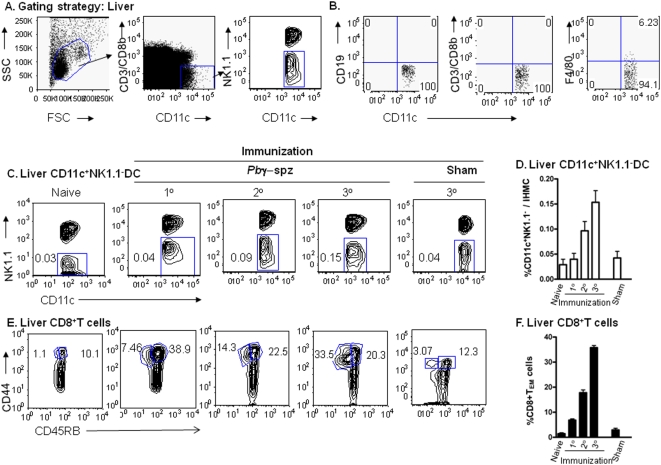
Both CD11c^+^NK1.1^−^ DC and CD8^+^ T_EM_ cells (CD44^hi^CD45RB^lo^) accumulate in the livers of *Pb* γ−spz-immunized mice. (A) HMNC were identified by FSC/SSC and after exclusion of CD3/CD8β^+^ T cells, CD11c^+^ cells are segregated into CD11c^+^NK1.1^+^ and CD11c^+^NK1.1^−^ populations. (B) Dot plots show the level or absence of B cells, T cells and macrophages within the gated CD11c^+^NK1.1^−^ population. (C) HMNC were isolated from individual C57BL/6 mice before and after prime and boost immunizations with *Pb* γ−spz or uninfected mosquito debris (sham). Cells were stained with a cocktail of mAbs and analyzed by flow cytometry for identification of CD11c^+^NK1.1^−^ DC and CD8^+^ T_EM_. Panels show representative contour plots of CD11c^+^NK1.1^−^ DC in the livers of naïve mice and livers of mice 6 days after the primary (1°), secondary (2°) and tertiary (3°) immunizations. The percentages of the CD11c^+^NK1.1^−^ DC in relation to the total HMNC/liver for each representative mouse are indicated in each panel. (D) The results show the mean %±SD of CD11c^+^NK1.1^−^ DC in total HMNC of naïve, *Pb* γ−spz-immunized and sham-immunized mice. (E) Panels show representative contour plots of CD8^+^ T_CM_ cells (CD44^hi^CD45RB^hi^ ) and CD8^+^ T_EM_ cells (CD44^hi^CD45RB^lo^) in the livers of naïve, *Pb* γ−spz-immunized and sham-immunized mice at the time-points indicated in (C). The percentages of the CD8+ T_EM_ and CD8+ T_CM_ cells in relation to the gated liver CD3^+^CD8^+^ T cells are indicated in the panels. (F) The results show the mean %±SD of CD8^+^ T_EM_ in the gated liver CD3^+^CD8^+^ T cells in naïve, *Pb* γ−spz-immunized and sham-immunized mice. The results are representative of three individual mice per group from three independent experiments.

**Figure 2:**
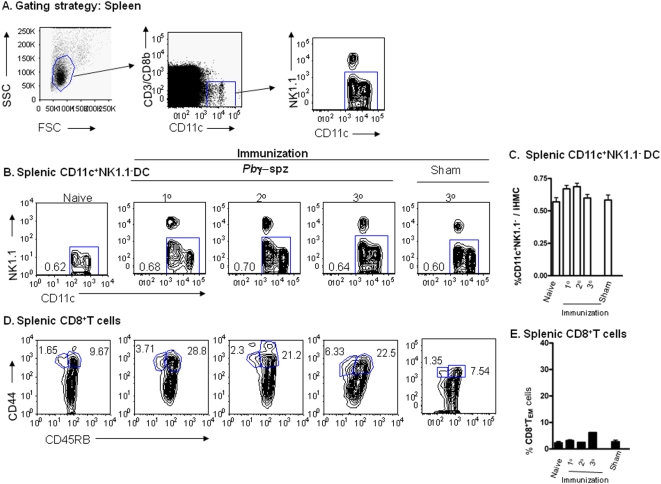
CD11c^+^NK1.1^−^ DC are constitutively present in the spleens of naïve mice but do not substantially increase following immunization. (A) CD11c^+^NK1.1^−^DC in *Pb*γ−spz-immune splenic MNC were identified according to the procedure described for HMNC in Fig 1. After exclusion of T cells, CD11c^+^ cells were segregated into CD11c^+^NK1.1^+^ and CD11c^+^NK1.1^−^ populations. Splenic mononuclear cells were isolated from individual mice before and after prime and boost immunizations with *Pb*γ−spz or uninfected mosquito debris (sham). Cells were stained with a cocktail of mAbs and NK1.1^−^ DC and CD8^+^ T_EM_ cells were identified by flow cytometry. (B) Panels show representative contour plots of DC in the spleens of naïve mice and in spleens of mice 6 days after either the 1°, 2° and 3° immunizations and in sham-immunized mice 6 days after the 3° immunization. The percentages of the CD11c^+^NK1.1^−^ DC in relation to the total SMNC/spleen for each representative mouse are indicated in each panel. (C) The results show the mean percentage ±SD of CD11c^+^NK1.1^−^ DC in total spleens of naïve mice, *Pb* γ−spz-immunized mice at day 6 after each immunization and in sham-immunized mice at day 6 after the 3° immunization. (D) Panels show representative contour plots of CD8^+^ T_CM_ cells and CD8^+^ T_EM_ cells in the spleens of naïve mice as well as of *Pb* γ−spz-immunized mice and sham-immunized mice at the same time-points described in (B). The numbers indicate the percentages of the T_CM_ and T_EM_ cells in the gated splenic CD3^+^CD8^+^ T cell population. (E) The results show the mean percentage ±SD of CD8^+^T_EM_ in the gated splenic CD3^+^CD8^+^ T cell population of naïve and immunized mice at day 6 after each immunization. Contour plots and bar graphs are representative of three individual mice per group in three independent experiments.

For the initial experiments we used flow cytometry to identify a population of CD11c^+^NK1.1^−^ DC in the liver and the spleen according to the gating strategy shown in Fig. 1A and Fig. 2A. To exclude all T cells and in particular CD8^+^ T cells (expressing CD8αβ dimers) as well as NK cells, intrahepatic mononuclear cells (IHMNC) and spleen cells were stained with anti-CD3/CD8β and anti-CD11c mAbs and the CD11c^+^CD3^−^CD8β^−^ population was further subdivided based on the expression of NK1.1 into CD11c^+^NK1.1^−^ DC and CD11c^+^NK1.1^+^ DC subsets. Further staining with anti- CD19, -CD3 and -F4/80 mAbs demonstrated that the resulting CD11c^+^NK1.1^−^ DC were negative for B cell, T cell and macrophage markers (Fig. 1B).

We next investigated whether repeated immunizations of C57BL/6 mice with *Pb*γ*−* spz led to the accumulation/expansion of the CD11c^+^NK1.1^−^DC populations in the liver. IHMNC were analyzed at baseline (naïve) and after each immunization for the presence of CD11c^+^NK1.1^−^ DC. Consistent with another published study [29], liver CD11c^+^NK1.1^−^ DC represented 0.02±0.019% (0.06×10^4^ cells) of the total IHMNC in naïve mice (Fig. 1C and D). Priming with *Pb* γ*−* spz increased the level of CD11c^+^NK1.1^−^ DC to 0.04±0.02% (0.14×10^4^ cells) of the total IHMNC; further increases up to 0.09±0.03% (0.75×10^4^ cells) and 0.15±0.04% (1.23×10^4^ cells) were observed after the first and second boost immunizations, respectively (Fig. 1C, D and Table 1). Prime and boost immunizations with uninfected mosquito debris (sham) resulted but in a negligible increase of CD11c^+^NK1.1^−^ DC in comparison to naïve CD11C^+^NK1.1^−^DC (Fig. 1C, D). Clearly, a population of CD11c^+^NK1.1^−^ DC that was nearly absent in the livers of naïve mice accumulated during the course of repeated immunizations with *Pb* γ*−* spz.

**Table 1:** Numbers of hepatic CD11c^+^NK1.1^−^ DC and cCD8α^+^DC in naïve and *Pb*γ-spz-immunized mice^a^

Immunization	HMNC (×10^6^)	NK1.1^−^ DC (×10^4^)	cCD8α^+^DC (×10^2^)
Naïve	1.80 (0.21)	0.06 (0.04)	0.04 (0.02)
Priming	3.30 (0.58)	0.14 (0.09)	0.56 (0.25)
Priming+1boost	7.60 (1.15)	0.75 (0.29)	3.80 (2.12)
Priming+2 boosts	8.30 (2.08)	1.23 (0.18)	9.32 (4.71)

a IHMNC were isolated from individual C57BL/6 mice before and after prime and boost immunizations with *Pb* γ−spz. Cells were stained with a cocktail of mAbs for identification of CD11c^+^NK1.1^−^ DC and cCD8α^+^DC as described in Materials and Methods and analyzed by flow cytometry. Data represent the mean±SD of the number of cells of three mice per group and are representative of three independent experiments.

Although hepatocytes serve as the primary venue for pre-erythrocytic stage development and replication of the mammalian *Plasmodia* spp, sporozoites also pass through the spleen. To determine whether multiple immunizations with *Pb* γ*−* spz might have caused an accumulation of DC in the spleen, we examined splenic cells at the same time points as IHMNC for the presence of CD11c^+^NK1.1^−^ DC. We detected CD11c^+^NK1.1^−^ DC in naïve spleens; however, they did not undergo any significant expansion during repeated immunizations with *Pb* γ*−* spz (Fig. 2 B, C).

### The increase in liver CD11c^+^NK1.1^−^ DC during immunization with *Pb* γ−spz coincides with the activation of liver CD8^+^ T cells

Although we previously documented the presence of CD8^+^T_EM_ (CD44^hi^CD45RB^lo^CD62L^lo^) and CD8^+^ T_CM_ (CD44^hi^CD45RB^hi^CD62L^int^ ) cells in the livers of mice immunized with *Pb* γ−spz [12], [30], we repeated these analyses along with that of liver DC to determine whether the induction of the liver CD8^+^ T cells was linked to the emergence of hepatic CD11c^+^NK1.1^−^ DC. As shown previously [12], [30], ∼1% of CD8^+^ T_EM_ cells was detected in the livers of naïve mice. Six days after priming, liver CD8^+^ T_EM_ cells increased significantly to 6.9±0.9% (1.4×10^5^ cells; p = 0.001) and subsequently, six days after the second and third immunizations, to 17.8±1.9% (5.2×10^5^ cells; p = 0.0001) and to 35.8±1.4% (11.1×10^5^ cells; p = 0.0001), respectively (Fig. 1E and F). Liver CD8^+^ T_EM_ cells increased negligibly (up to 3%) after the third immunization with uninfected mosquito debris (sham) (Fig. 1E and F). Liver CD8^+^ T_CM_ cells also increased following immunization with *Pb* γ−spz, but because CD8^+^ T_CM_ were already present at an elevated level (∼10%) in the livers of naïve mice, the only significant expansion was seen after the first immunization when the levels increased to ∼40% (p = 0.005) (Fig. 1E). Collectively, these results show that the expansion of liver CD8^+^ T_EM_ cells in particular was concurrent with the accumulation of CD11c^+^NK1.1^−^ DC in the livers of *Pb* γ−spz-immunized mice, thus implying a key role for CD11c^+^NK1.1^−^ DC in the presentation of liver-stage Ags to liver CD8^+^ T cells that mediate protection induced by *Pb* γ-spz.

Overall, the percentages of CD8^+^ T_EM_ cells in the spleens were lower than those in the livers of the *Pb* γ−spz immunized mice and similar to our previously published studies [12], [30], we observed no significant accumulation of CD8^+^ T_EM_ cells in the spleens of the mice after repeated immunizations with *Pb* γ−spz. (Fig. 2D, E). These observations strengthen our hypothesis that the presence of CD11c^+^NK1.1^−^ DC in the liver was coincident with the activation of liver CD8^+^ T_EM_ and T_CM_ cells after immunization with *Pb* γ−spz.

### cCD8α^+^ DC numbers are low in the livers of naïve mice but increase following immunization with *Pb* γ-spz

**Figure 3:**
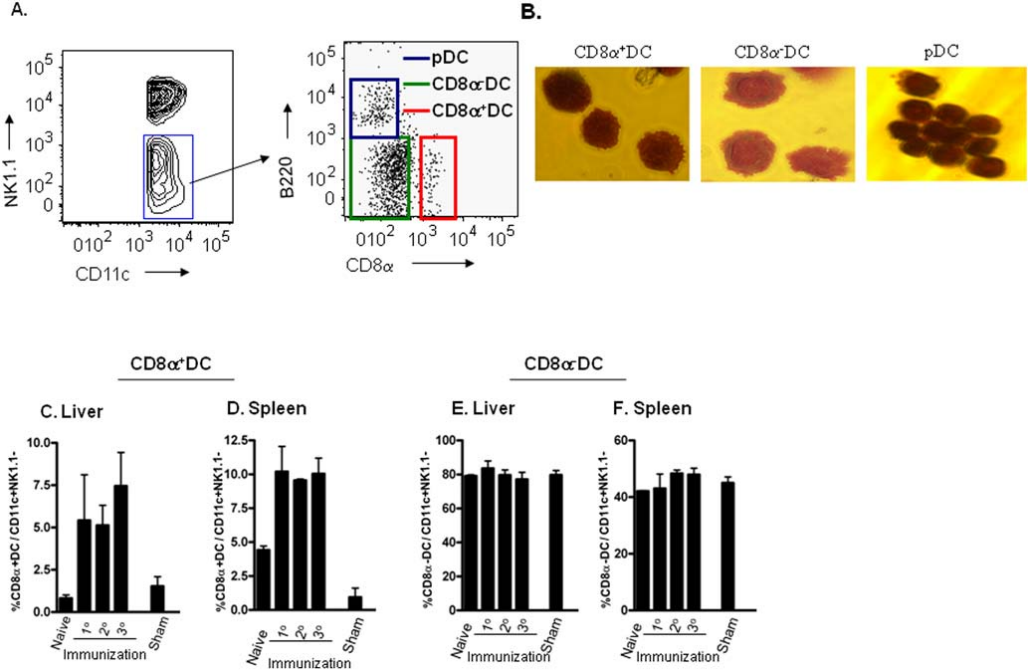
cCD8α^+^ DC, relatively absent in the livers of naïve mice, are induced after prime-boost immunizations with *Pb*γ-spz. (A) Hepatic CD11c^+^NK1.1^−^ DC (see Fig. 1) were stained with Abs specific for B220 and CD8α and analyzed by flow cytometry to reveal 3 subpopulations of DC . (B) HMNC isolated from livers of *Pb*γ-spz-fully immunized mice 6 days after the 3° immunization were incubated with a cocktail of biotinylated microbeads to deplete T cells, B cells, NK cells, granulocytes and macrophages as described in Materials and Methods. DC subpopulations were further isolated from the enriched CD11c^+^NK1.1^−^ population by positive magnetic selection for cCD8α^+^DC and pDC and by negative selection for CD8α^−^DC, as described in Materials and Methods. Panels show photographs at 100× of Giemsa stained cytospins of cCD8α^+^DC, cCD8α^−^DC and pDC. (C and E) HMNC and (D and F) splenic MNC were isolated from individual naïve mice and from individual mice at 6 days after 1°, 2° and 3° immunizations with *Pb* γ−spz and after 3° immunizations with uninfected mosquito debris (sham). Cells were stained with a cocktail of mAbs for identification of DC subpopulations as described in (A). Bar graphs show the mean % ± SD of the cCD8α^+^DC (C and D) and cCD8α^−^DC (E and F) in the gated CD11c^+^NK1.1^−^ cells. Data are representative of three individual mice per group at each time-point in two independent experiments.

It has been reported that splenic and liver DC represent heterogeneous populations [31], [32] and based on the expression of cell surface markers they are divided into three major subsets: cCD8α^+^ DC and cCD8α^−^ DC as well as pDC. Moreover, these various DC subpopulations differ in their capacity to activate T cells [33], [34]. There is abundant evidence especially from studies of immune responses to viral infections [2] that cCD8α^+^ DC are the main APC responsible for cross-priming of CD8^+^ T cells. Similarly, liver cCD8α^+^ DC have been shown to produce pro-inflammatory cytokines and prime peptide-specific CD8^+^ T cells during Salmonella infection [35]. Therefore, we asked whether cCD8α^+^DC were present in the expanded population of CD11c^+^NK1.1^−^ DC that accumulated in the livers of *Pb* γ-spz immunized mice. The CD11c^+^NK1.1^−^ DC were gated as described in Fig. 1A and then evaluated for the presence of CD8α^+^B220^−^ (cCD8α^+^DC), CD8α^−^B220^−^ (cCD8α^−^DC) and CD8α^−^B220^+^ (pDC) (Fig. 3A).

Cytospin preparations of the purified DC subpopulations were stained with Giemsa and examined by microscopy. cCD8α^+^DC possessed an abundant cytoplasm and displayed the characteristic dendrites whereas cCD8α^−^DC were characterized by an abundant cytoplasm and an irregular surface. Plasmacytoid DC displayed a round, smooth surface and an oval nucleus (Fig. 3B).

The relative percentages of cCD8α^+^DC were determined in naïve mice and in *Pb* γ-spz immunized mice 6 days after each successive immunization. As can be seen from Fig. 3C, cCD8α^+^ DC represented 0.8±0.3% of the CD11c^+^NK1.1^−^ population in naïve mice. After priming, we observed an increase of up to 5.4±4.7%; subsequently, after the second and third immunizations with *Pb* γ-spz the cells remained relatively stable at 5.1±2.0% and 7.4±1.9%, respectively. The same trend was observed when we considered the total number of cCD8α^+^ DC (Table 1). The percentage of cCD8α^+^ DC following three immunizations with mosquito debris (sham) was only slightly elevated in comparison to naïve control mice and did not exceed 1.5±0.8% of the CD11c^+^NK1.1^−^ cells (p = 0.3). Parallel analysis of splenic CD11c^+^NK1.1^−^ DC showed the presence of cCD8α^+^ DC at 4.4%±0.5% in naïve mice; after priming there was an increase up to 10.2±3.2%, but the levels remained relatively constant at 9.6±0.2% and 10.1±1.9% of the CD11c^+^NK1.1^−^ DC following the second and the third immunizations, (Fig. 3D). Similar trends were observed with respect to total numbers of splenic DCs (Table 2). There was no increase in the percentage of cCD8α^−^ DC in either the liver or spleen following immunization with *Pb* γ-spz (Fig. 3E and F).

**Table 2:** Numbers of splenic CD11c^+^NK1.1^−^ DC and cCD8α^+^DC in naïve and *Pb*γ-spz-immunized mice^a^

Immunization	SMNC (×10^6^)	NK1.1^−^ DC (×10^4^)	cCD8α^+^DC (×10^3^)
Naïve	77.41 (4.21)	11.32 (1.45)	4.97 (0.44)
Priming	84.67 (5.69)	14.21 (3.51)	13.97 (2.59)
Priming+1 boost	74.33 (6.03)	16.31 (2.60)	15.59 (2.68)
Priming+2 boosts	85.33 (5.69)	15.64 (1.62)	15.51 (1.56)

a SMNC were isolated from individual C57BL/6 mice before and after prime and boost immunizations with *Pb* γ−spz. Cells were stained with a cocktail of mAbs for identification of CD11c^+^NK1.1^−^ DC and cCD8α^+^DC as described in Materials and Methods and analyzed by flow cytometry. Data represent the mean±SD of the number of cells of three mice per group and are representative of three independent experiments.

**Figure 4:**
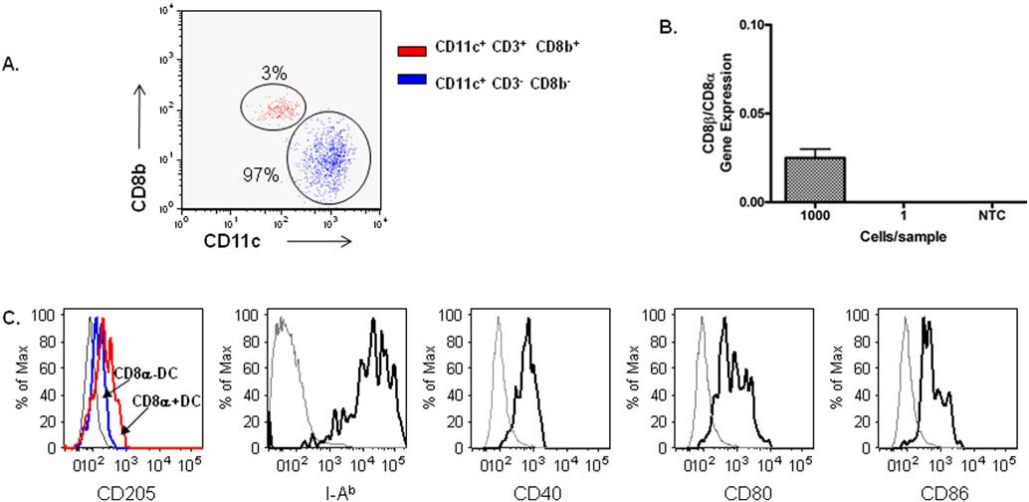
CD8β mRNA is absent in purified liver cCD8α^+^DC. HMNC were pooled from livers of *Pb*γ-spz-fully immunized mice 6 days after the 3° immunization and were incubated with a cocktail of biotinylated microbeads to deplete T cells, B cells, NK cells, granulocytes and macrophages as described in Materials and Methods. cCD8α^+^DC were further isolated from the enriched CD11c^+^NK1.1^−^ population by positive magnetic selection as described in Materials and Methods. Staining with anti-CD8α, CD3, CD8b and CD11c was performed on permeabilized cells to reveal both the surface and the intracellular presence of these markers. (A) Dot plot show the relative % of T cells (red) and cCD8α^+^DC (blue) within the purified cCD8α^+^DC population. (B) Two-step quantitative real-time PCR was performed on RNA isolated from magnetic-bead purified liver cCD8α^+^DC (described in A). Ratio of CD8β/CD8α gene expression was calculated using standard curves for each gene. Measurements were done in duplicates in wells containing 1000, 1, 0.1 cells/well, or non-template control (NTC). Representative results of one out of two experiments are shown. (C) Histogram plots show expression of DEC 205, I-A^b^ and costimulatory molecules on the cCD8α^+^DC population (black lines). Grey lines represent staining of the isotype controls.

To confirm that the liver cCD8α^+^ DC induced following immunization with *Pb* γ-spz were not CD8^+^ T cells that have lost or down-regulated the CD3 marker, we subjected the purified liver cCD8α^+^ DC (Materials and Methods) to two separate analyses: (1) flow cytometry for both surface and intracellular expression of CD3, CD8β and CD11c and (2) quantitative RT- PCR for the presence of CD8β mRNA. According to the flow cytometry results, liver cCD8α^+^ DC were >97% pure (Fig. 4A) and this population was negative for CD8β mRNA when assayed at 1 cell per well. (Fig. 4B). These observations not only confirm that the cCD8α^+^ DC is a subset of the liver CD11c^+^NK1.1^−^DC, but they demonstrate a significant accumulation of cCD8α^+^ DC in the livers of *Pb*γ-spz-immunized mice (Fig. 3C). We also characterized the liver cCD8α^+^DC for the expression of CD205, MHC and co-stimulatory molecules as they have been shown [36] to be expressed on this subset of DC.We found that indeed cCD8α^+^ DC expressed higher levels of CD205 than CD8α^−^DC (MFI = 252 vs MFI = 166, respectively). The cCD8α^+^ DC also exhibited high expression of MHC class I (MFI = 11×10^3^) and class II (MFI = 14×10^3^) and moderate levels of CD40 (MFI = 545), CD80 (MFI = 667) and CD86 (MFI = 512) (Fig. 4C).

### Liver cCD8α^+^ DC are the primary inducers of CD8^+^ T cell activation

**Figure 5:**
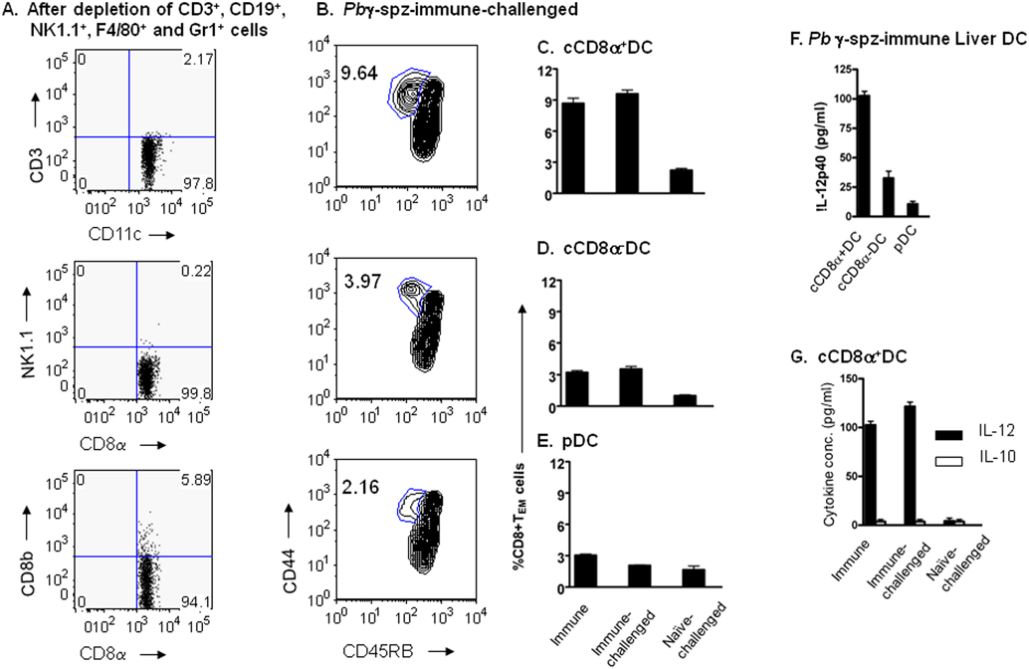
Hepatic cCD8α^+^ DC from *Pb*γ-spz-immunized and *Pb*γ-spz-immunized and challenged mice mediate *in vitro* activation of naïve CD8^+^ T cells. HMNC were pooled from livers of *Pb*γ-spz-fully immunized mice (n = 20) 6 days after the 3° immunization and were incubated with a cocktail of biotinylated microbeads to deplete T cells, B cells, NK cells, granulocytes and macrophages as described in Materials and Methods. DC subpopulations were further isolated from the enriched CD11c^+^NK1.1^−^ population by positive magnetic selection for cCD8α^+^DC and pDC and by negative selection for CD8α^−^DC, as described in Materials and Methods. (A) Dot plots show the relative % of T cells and NK1.1 cells within the gated CD11c^+^CD8α^+^DC. (B) cCD8α^+^ DC, cCD8α^−^ DC and pDC were purified from CD11c^+^NK1.1^−^ DC isolated from pooled livers either 6 days after 3° immunization or 3 days after the challenge of *Pb*γ-spz-immunized as well as naïve mice . CD8^+^ T cells were isolated from the spleens of naïve mice using magnetic beads. Liver DC subpopulations and splenic CD8^+^ T cells were co-cultured at a ratio of 1 DC : 2 CD8^+^T cells for 4 days. Culture supernatants were harvested and analyzed for IL-10 and IL-12p40 by ELISA. Cells were harvested, stained with a cocktail of mAbs and the % of CD3^+^CD8^+^CD45RB^lo^CD44^hi^ cells (CD8^+^ T_EM_) was analyzed by flow cytometry. Results show representative contour plots of CD8^+^T cells co-cultured with cDC and pDC subpopulations from *Pb* γ-spz-immunized-challenged mice. Bar graphs show the mean percentage±SD of CD8^+^ T_EM_ in the gated CD3^+^CD8^+^ T cell population after co-culture with (C) cCD8α^+^ DC, (D) cCD8α^−^ DC and (E) pDC each isolated from *Pb*γ-spz-fully-immunized, *Pb*γ-spz-immunized-challenged and naïve-challenged mice. The experiments were performed twice yielding similar results. (F) IL-12p40 production in the culture supernatants of CD8^+^ T cells with the liver DC populations from the *Pb*γ-spz-immunized mice. The results expressed as pg/ml (mean±SD) represent data from two representative experiments. (G) cCD8α^+^DC were isolated from pooled livers of *Pb*γ-spz-fully-immunized mice, *Pb*γ-spz-immunized and challenged mice or naïve-challenged mice and co-cultured with purified naïve splenic CD8^+^ T cells for 4 days. Supernatants were harvested and analyzed for IL-12p40 and IL-10 by ELISA.

**Figure 6:**
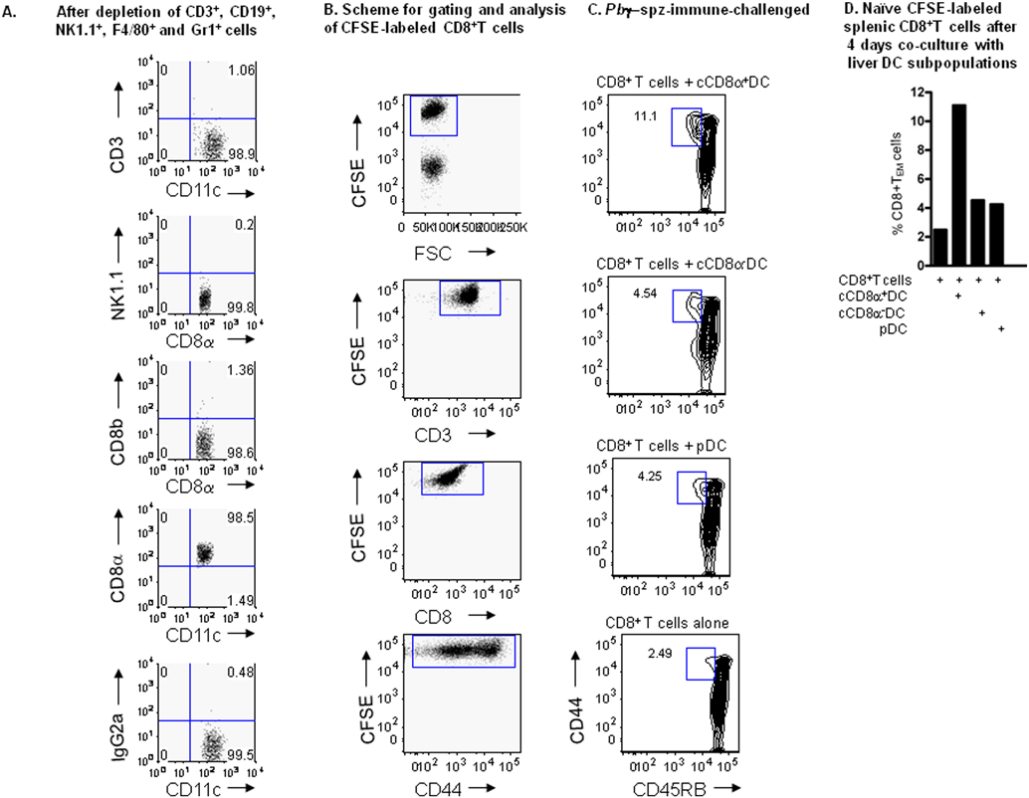
Hepatic cCD8α^+^ DC from *Pb*γ-spz-immunized and challenged mice mediate *in vitro* activation of naïve CD8^+^ T cells. HMNC were pooled from livers of *Pb*γ-spz-fully immunized and challenged mice (n = 18) and were incubated with a cocktail of biotinylated microbeads to deplete T cells, B cells, NK cells, granulocytes and macrophages as described in Materials and Methods. DC subpopulations were further isolated from the enriched CD11c^+^NK1.1^−^ population by positive magnetic selection for cCD8α^+^DC and pDC and by negative selection for CD8α^−^DC, as described in Materials and Methods. (A) Dot plots show the relative % of T cells and NK1.1 cells within the purified cCD8α^+^DC population. (B) CD8^+^ T cells were isolated from the spleens of naïve mice using magnetic beads and labeled with 2 µM CFSE. Dot plots show the gating scheme for the analysis of CFSE-labeled CD3^+^CD8^+^T cells for expression of the CD45RB^lo^CD44^hi^ phenotype. (C) cCD8α^+^ DC, cCD8α^−^ DC and pDC were purified from CD11c^+^NK1.1^−^ DC isolated from pooled livers 3 days after the challenge of *Pb*γ-spz-immunized mice. Liver DC subpopulations and CFSE-labeled splenic CD8^+^ T cells were co-cultured at a ratio of 1 DC : 2 CD8^+^T cells for 4 days. Cells were harvested, stained with a cocktail of mAbs and the % of CD3^+^CD8^+^CD45RB^lo^CD44^hi^ cells (CD8^+^ T_EM_) was analyzed by flow cytometry. Results show contour plots of CD8^+^T cells co-cultured with cDC and pDC subpopulations. (D) Bar graphs show the percentage of CD8^+^ T_EM_ in the gated CD3^+^CD8^+^ T cell population after co-culture with cCD8α^+^ DC, cCD8α^−^ DC and pDC each isolated from *Pb*γ-spz-immunized-challenged mice.

Results from two preliminary experiments showed that CD8^+^ T cells acquired the CD44^hi^CD45RB^lo^ activation phenotype upon *in vitro* and *in vivo* exposure to CD11c^+^NK1.1^−^ DC purified from the livers of *Pb γ*-spz-immunized mice. Because cCD8α^+^DC are considered as the primary APC involved in cross-presentation of Ags to CD8^+^ T cells, we proceeded to test the capacity of liver cCD8α^+^DC from Pb γ-spz-immunized mice to serve as APC for the activation/differentiation of naïve CD8^+^ T cells and, by extension, to confirm the involvement of DC in the development of protective immunity. CD11c^+^NK1.1^−^ DC isolated from pooled livers were further enriched by magnetic depletion using a cocktail of mAbs (anti-CD3, -CD19, -F4/80, -NK1.1, -Gr-1). cCD8α^+^ DC and pDC cells within the enriched CD11c^+^NK1.1^−^ DC were each obtained by positive selection whereas the cCD8α^−^ DC were obtained by negative selection. The purity of the enriched cCD8α^+^ DC ranged between 94–98% based on CD11c, CD8β, CD3 and NK1.1 expression (Fig. 5A and 6A). The purity of the cCD8α^−^ DC and pDC was also >90%. We co-cultured negatively selected CD8^+^ T cells from naïve mice with either cCD8α^+^ DC, cCD8α^−^ DC or pDC derived from the livers of mice immunized with *Pb* γ-spz or mice immunized with *Pb* γ-spz and challenged with infectious sporozoites and analyzed the CD8^+^ T cells on day 4 of the co-culture for the expression of the CD44^hi^CD45RB^lo^activation phenotype. As is evident from the results in Fig. 5B–E, cCD8α^+^ DC derived from the livers of either fully immunized or fully immunized-challenged mice were 3–4 fold more effective than cCD8α^−^ DC and pDC in inducing CD8^+^ T cells to acquire the CD44^hi^CD45RB^lo^ activation phenotype.

As was shown in Figs. 4, 5A and 6A, approximately 2–5% CD8^+^ T cells were present within the purified cCD8α^+^DC subpopulation. To minimize the potential effect of these contaminating immune CD8^+^ T cells in the co-cultures of naïve CD8^+^ T cells and cCD8α^+^DC, we labeled naïve CD8^+^T cells with CFSE before adding them to DC. At the conclusion of a 4-day culture, the CFSE-labeled CD8^+^ T cells were analyzed for the expression of the CD44^hi^CD45RB^lo^ phenotype. The gating scheme for the analysis of the CFSE-labeled splenic CD3^+^CD8^+^T cells is shown in Fig. 6B. Similar to the findings shown in Fig. 5, these results indicate that cCD8α^+^ DC derived from the livers of fully immunized-challenged mice were 4-fold more effective than cCD8α^−^ DC and pDC in inducing CD8^+^ T cells to acquire the CD44^hi^CD45RB^lo^ phenotype (Fig. 6C and D).

### cCD8α^+^ DC are the major IL-12 producers following challenge of *Pb* γ-spz-immunized mice

IL-12 is a key cytokine promoting the activation of CD8^+^ T cells [37], therefore, we evaluated the different liver DC subpopulations to assess if the superior APC properties of the cCD8α^+^ DC might be related to a greater capacity for producing IL-12. Levels of IL-12p40 were measured in day 4 supernatants from co-cultures of naïve CD8^+^ T cells with the three DC subpopulations from fully-immunized mice (Fig. 5F) and the results revealed that the cCD8α^+^ DC were the highest (>100 pg/ml), the cCD8α^−^ DC were the intermediate (<50 pg/ml) and pDC were the lowest (<10 pg/ml) producers of IL-12p40. IL-10 was not detected in any of the culture supernatants (data not shown). It should be pointed out that PCR analysis of cytokine specific mRNA isolated from each DC subset confirmed the ELISA results (data not shown).

We also compared the levels of IL-12p40 and IL-10 in the day-4 supernatants co-cultures of naïve CD8^+^ T cells with the cCD8α^+^ DC subpopulations from fully-immunized or fully-immunized and challenged or naïve-challenged mice. As shown in Fig. 5G, high levels of IL-12p40 were detected in the culture supernatants with cCD8α^+^ DC from fully-immunized or from fully-immunized and challenged mice. In contrast, IL-12p40 was not detected in the supernatants of co-cultures containing cCD8α^+^ DC from naïve-challenged mice. IL-10 was not detected in any of the culture supernatants from any of the groups tested.

### Activation of CD8^+^ T cells by liver cCD8α^+^ DC from *Pb* γ−spz-immunized mice is MHC class I- and IL-12-dependent

Ag-specific activation of CD8^+^ T cells is mediated via the *sine qua non* ligation of the TCR by peptide:MHC class I complexes and it also requires secondary signals delivered via co-stimulatory molecules [38] and tertiary signals from cytokines [39]. To test whether this CD8^+^ T cell activation was mediated by the occupancy of the TCR by MHC:peptide complexes and if it was also dependent upon IL-12, we established parallel co-cultures consisting of liver or spleen cCD8α^+^ DC from mice fully immunized with *Pb* γ-spz and liver or splenic CD8^+^ T cells from naïve mice in the presence or absence of either anti-MHC class I mAb or anti-IL-12 mAb. After 4 days, the acquisition of the CD44^hi^CD45RB^lo^ phenotype by CD8^+^ T cells was analyzed by flow cytometry and in parallel, culture supernatants were assayed for IFN-γ.

**Figure 7:**
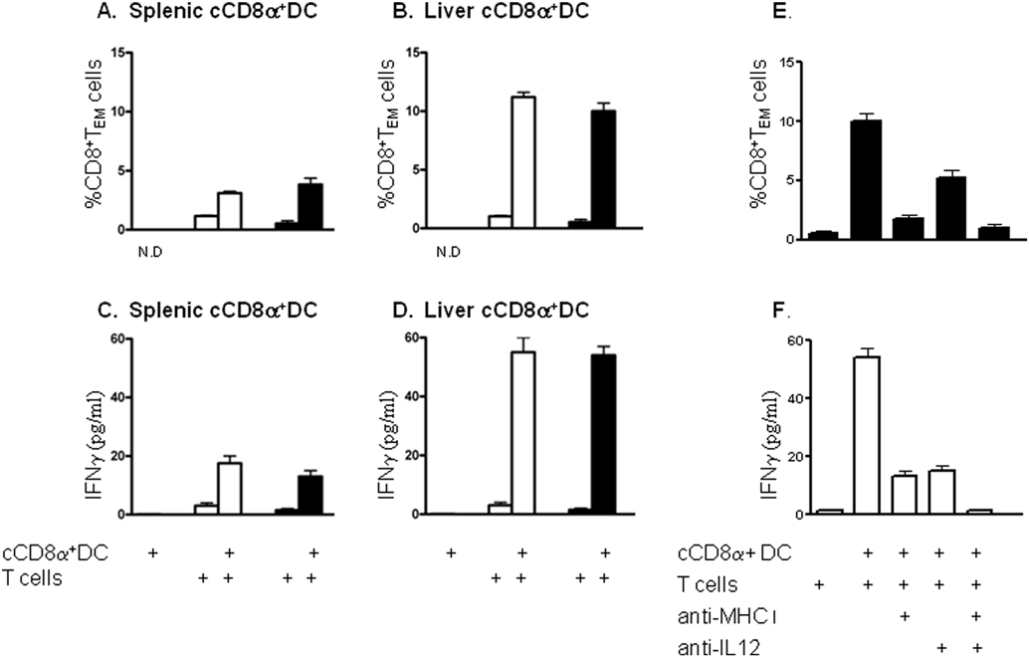
Liver cCD8α^+^ DC are more efficient than splenic cCD8α^+^ DC in inducing differentiation of and IFN-γ production by CD8^+^ T cells. Differentiation and induction of CD8^+^ T cell function is MHC class I- and IL-12 dependent. HMNC or splenic MNC were prepared after the 3° immunization as described in Fig. 3. CD8α^+^ DC were purified as described in Figs 1 and 3. Splenic (A and C) or hepatic (B and D) cCD8α^+^ DC were co-cultured for 4 days either alone or with purified CD8^+^ T cells from the livers (open bars) or spleen (filled bars) of naïve mice. Cells were harvested, stained with the appropriate mAb and analyzed by flow cytometry. Culture supernatants were analyzed for IFNγ by ELISA. (A and B) Results show the mean % of CD8^+^ T_EM_ in the gated CD3^+^CD8^+^ T cell population and (C and D) the amount of IFNγ in the culture supernatant. Data are representative of two individual experiments. (E and F) Liver cCD8α^+^ DC were co-cultured with naïve splenic CD8^+^ T cells in the presence or absence of anti-IL-12 (clone C17.1) and/or anti-MHC class I (clone 28-8-6) mAbs for 4 days. (E) Results show the mean % of CD8^+^ T_EM_ in the gated CD3^+^CD8^+^ T cell population and (F).the amount of IFNγ. Data is representative of two individual experiments.

The APC activity of the liver cCD8α^+^ DC from *Pb* γ-spz immunized mice was markedly superior to that of the same subpopulation of splenic DC, regardless of the source of naïve CD8^+^ T cells. Whereas the splenic DC induced approximately 5% of CD8^+^ T cells to express the CD44^hi^CD45RB^lo^ phenotype (Fig. 7A), the liver DC induced at least 2–3-fold higher percentage of these cells (Fig. 7B). The same superior APC activity of liver DC was also indicated by the fact that the level of IFN-γ was nearly three-fold higher (60 pg/ml vs 20 pg/ml) (Fig. 7D) in the supernatants of co-cultures of CD8^+^ T cells and liver cCD8α^+^ DC as compared to co-cultures with splenic cCD8α^+^ DC (Fig. 7C **)**. IFNγ was not detected in the supernatants when cCD8α^+^ DC from either source were cultured alone (Fig.7 C, D).

The capacity of immune liver cCD8α^+^ DC to induce CD8^+^ T cells to acquire an activation phenotype and produce IFN-γ was reduced by nearly 70% in the presence of anti-MHC class I mAb. Although the inclusion of anti-IL-12 mAb had a somewhat lower inhibitory effect than anti-MHC class I mAb, it also significantly diminished both responses (Fig. 7E and F). Addition of both blocking antibodies to the co-culture abrogated the activation of CD8^+^ T cells by reducing both the numbers of CD44^hi^CD45RB^lo^ CD8^+^ T cells and IFN-γ production to nearly control levels (response with CD8^+^ T cells alone), thus suggesting an additive or even synergistic effect of TCR ligation by MHC class I:peptide complexes and IL-12 signaling in the activation of the CD8^+^ T cells by liver cCD8α^+^ DC.

### Adoptively transferred liver CD11c^+^NK1.1^−^ DC obtained from *Pb* γ−spz-immunized mice confer protection against liver-stage infection

**Figure 8:**
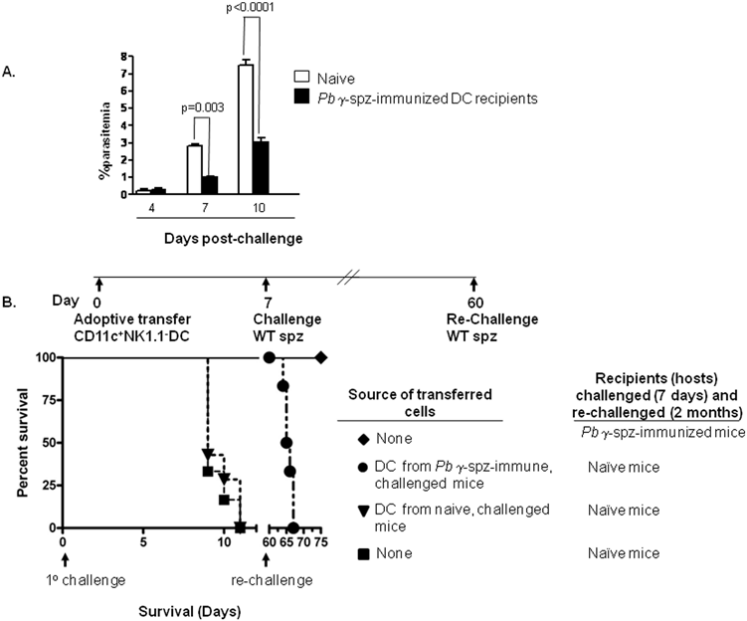
Liver CD11c^+^NK1.1^−^ cells from *Pb*γ-spz-immunized mice induce protection against infectious sporozoites. (A) 1×10^6^ CD11c^+^NK1.1^−^ cells, isolated from the livers of *Pb*γ-spz-fully-immunized mice 6 days after the 3° immunization, were adoptively transferred (i.v.) into naïve recipients. Seven days later the adoptively transferred recipients (n = 3) as well as naïve infectivity control mice (n = 3) were challenged with 10K infectious sporozoites. The results show the level of parasitemia assessed in each individual mouse and expressed as the mean of parasitemia per mice/group at 4, 7 and 10 days following challenge. (B) 1×10^6^ hepatic CD11c^+^NK1.1^−^ cells, purified as described in (A) were isolated from *Pb*γ-spz-immunized-challenged mice and from naïve-challenged mice and adoptively transferred into naïve syngeneic recipients (n = 13) that were challenged 7 days later with either 250 (n = 6) or 1000 (n = 7) infectious sporozoites. The protected group (250 sporozoites) were re-challenged 60 days later along with another group of naïve infectivity control mice (n = 3). *Pb* γ-spz-immunized mice (n = 3), used as positive controls, were sterily protected at challenge and re-challenge. Parasitemia and survival were evaluated from day 2 post-challenge.

It has been reported that adoptive transfer of bone marrow-derived DC pulsed with *Py* CS protein peptide significantly reduces parasitemia in Balb/c mice challenged with *Py* sporozoites [15]. Based on our preliminary observations that liver CD11c^+^NK1.1^−^ DC from *Pb γ-*spz immunized mice were able to activate CD8^+^ T cells both *in vitro* and *in vivo* (data not shown), we hypothesized that adoptive transfer of these DC into naïve mice might confer protection or at least reduce the level of parasitemia in sporozoite-challenged recipients. In an initial experiment, 1×10^6^ liver CD11c^+^NK1.1^−^ DC from *Pb γ-*spz−immunized mice were adoptively transferred (i.v.) into naïve recipients and this group as well as a control group not receiving any DC were subsequently challenged (i.v.) with 10K infectious *Pb* sporozoites. The results showed that even though the transferred hepatic CD11c^+^NK1.1^−^ DC significantly decreased parasitemia (Fig. 8A) and prolonged survival following infectious challenge, they were unable to confer sterile protection. Because 10K infectious sporozoites constitute a very high challenge dose, we asked whether protection could be achieved against a lower challenge dose. Thus, in the next set of experiments CD11c^+^NK1.1^−^ DC from the livers of *Pb γ-*spz immunized-challenged or naïve-challenged mice were transferred separately into naïve recipients that were subsequently challenged with 250 sporozoites. As shown in Fig. 8B, recipients of hepatic CD11c^+^NK1.1^−^ DC from *Pb γ-*spz-immunized-challenged mice were sterilely protected when challenged with 250 infectious sporozoites. However, protection was lost upon re-challenge at 6 months with 250 infectious sporozoites, implying that sterile protective immunity induced by the transferred CD11c^+^NK1.1^−^ DC was short-lived. Notably, hepatic CD11c^+^NK1.1^−^ DC from naïve-challenged mice did not confer protection even against the low-dose challenge. Collectively, these results strongly support our contention that liver CD11c^+^NK1.1^−^ DC are actively involved in mediating protective immunity induced by *Pb* γ-spz and that their protective function depends upon their capacity to present Ag to liver CD8^+^ T cells that leads to the induction of effector cells against infectious sporozoites.

## Discussion

In the present study we demonstrated that CD8α^+^CD11c^+^NK1.1^−^ DC, although infrequent in the livers of naïve mice, increased in number in the livers of mice undergoing prime-boost immunizations with *Pb* γ-spz. The numbers as well as percentages of the liver cCD8α^+^DC increased significantly after priming and were maintained following boost immunizations, but declined after challenge with infectious sporozoites. The emergence of liver DC was concurrent with the appearance of hepatic CD8^+^ T_EM_ cells (CD44^hi^CD45RB^lo^), that in agreement with our previous findings [12], [30], accumulate slowly, with the peak response occurring only after the 3^rd^ immunization. This temporal relationship between the CD11c^+^NK1.1^−^ DC and CD8^+^ T cells suggests that liver DC serve as key *Pb* liver-stage Ag-presenting cells that induce liver CD8^+^ T cells to differentiate into the major effector T cells that confer protection against infectious sporozoite challenge. This contention is further supported by our demonstration that liver cCD8α^+^ DC from either *Pb* γ-spz-immunized or *Pb* γ-spz-immunized-challenged mice induced CD8^+^ T cells from naïve mice to express the CD44^hi^CD45RB^lo^ activation phenotype and to produce IFN-γ in an *in vitro* system. Most importantly, adoptive transfer of purified liver CD11c^+^NK1.1^−^ DC isolated from *Pb* γ-spz immunized donors conferred protective immunity to congenic recipient mice challenged with infectious sporozoites.

Like infectious Plasmodia sporozoites, *Pb* γ-spz also invade hepatocytes but they undergo developmental arrest at the liver-stage. The aborted forms of liver-stage parasites are thought to provide a repository of liver-stage Ags needed for the induction and the maintenance of sterile and long lasting protective immunity [28]. However, the mechanisms involved in the processing of these Ags and their presentation to T cells remain to be fully elucidated. Recent studies [22], [40] suggest that infection with either *Pb* γ-spz or genetically-attenuated Plasmodia sporozoites causes hepatocytes to undergo apoptosis, a process that is currently viewed as a potent trigger of host innate and adaptive immune responses [41], [42]. Therefore, it is reasonable to suggest that the attenuated sporozoite-infected apoptotic hepatocytes provide a rich repertoire of liver-stage Ags that are taken up by the liver DC and cross-presented to CD8^+^ T cells in the liver.

Numerous studies [2], [3], [25] have shown that cCD8α^+^ DC are the predominant subpopulation of DC that mediate cross-presentation of cell-associated bacterial and viral Ags for the activation of CD8^+^ T cells. Data from the present study demonstrate for the first time that cCD8α^+^ DC indeed were represented in the hepatic CD11c^+^NK1.1^−^ DC of *Pb* γ-spz-immunized mice. Although nearly absent in the livers of naïve mice, this subset increased significantly after the priming dose of *Pb* γ-spz and it was the most effective APC that induced CD8^+^ T cells to both acquire the CD44^hi^CD45RB^lo^ activation phenotype and to produce IFN-γ *in vitro.* On the basis of these observations, we propose that liver cCD8α^+^ DC cross-present *P. berghei* liver-stage Ags derived from apoptotic *Pb*-infected hepatocytes to liver CD8^+^T cells.

Based on their percentages and numbers, the cCD8α^+^ DC were not the dominant subset in the livers of *Pb* γ-spz immunized or *Pb* γ-spz immunized-challenged mice. Nevertheless, the liver cCD8α^+^ DC exhibited superior APC activity relative to the other two subsets of liver DC and to splenic cCD8α^+^ DC derived from the same animals. The splenic cCD8α^+^ DC remained at a steady level (∼10%) during the entire prime-boost immunization with *Pb* γ-spz and their capacity to activate CD8^+^ T cells from naïve mice to express the CD44^hi^CD45Rb^lo^ phenotype and to produce IFN-γ was 4-fold lower than that of liver cCD8α^+^ DC. This dichotomy between APC activities of liver and spleen cCD8α^+^ DC may be explained by the fact that *Pb* γ-spz primarily invade and undergo a partial development within hepatocytes. Consequently, the availability of liver-stage Ags for cross-presentation would likely be higher in the liver than the spleen. Thus, the site of the induction of CD8^+^ T cells (liver versus spleen) might be related to the local availability of Ags for presentation to the responding CD8^+^ T cells.

Infectious sporozoites develop into liver merozoites that are released into the blood stream to infect red blood cells and thus, initiate clinical malaria. Challenging naive mice with infectious sporozoites also led to an accumulation of cCD8α^+^ DC in the liver; however the APC activity of these cells was rather ineffective in comparison to that of liver cCD8α^+^ DC from *Pb*γ-spz immunized or *Pb*γ-spz immunized and challenged mice. Several mechanisms may be proposed to explain the difference between the two populations of liver cCD8α^+^ DC. First, two recent reports [23], [24] showed that, in contrast to attenuated sporozoites, infectious *Pb* sporozoites do not induce infected hepatocytes to undergo apoptosis and hence are able to complete their differentiation into liver-stage merozoites that are released into the blood stream without prior alarming or inducing the immune system. Consequently, there is likely a paucity of available early liver stage Ags in mice with active liver stage infection. Secondly, our previous results demonstrated that MHC class I molecules are down-regulated on both Kupffer cells (KC) [43] and liver DC (unpublished data) 24–48 hrs after the challenge of naive mice. The decreased intensity of MHC class I on the surface of KC leads to a diminution in their APC function [43], [44]. The reduction of MHC class I on cCD8α^+^ DC in infected animals (data not shown) also might have reduced their capacity to present liver-stage Ag to CD8^+^ T cells. Finally, cCD8α^+^ DC from livers of naïve challenged mice do not produce detectable levels of IL-12 and this also could have compromised their capacity to activate CD8^+^ T cells. These results extend the recently published observations that IL-12-producing DC are found only in mice infected with a non-lethal strain of *P. yoelii* but not in mice infected with the lethal strain [45]. It should also be noted that in contrast to our observations, a recently published study showed that human pDC that produce IFN-α following viral infection also were able to cross-prime CD8^+^ T cells [46].

DC-mediated differentiation of Ag-specific CD8^+^ T cells involves the requisite signal 1 delivered through the ligation of the TCR by peptide-MHC class I complexes as well as the necessary provision of co-stimulation (signal 2) and pro-inflammatory cytokines such as IL-12 (signal 3). Although the characterization of *Pb* liver-stage Ags is in progress, the exact nature of these Ags is currently unknown. Consequently, we were unable to use defined tetramers or transgenic CD8^+^ T cells to verify the *Pb* Ag specificity of the *Pb* γ-spz-immune-CD8^+^ T cells. However, the Ag-specificity of the responding CD8^+^ T cells is strongly implied by the observation that IFN-γ production and the expression of the CD44^hi^CD45RB^lo^ on CD8+ T cells were abrogated by nearly 70% by anti-MHC class I mAb. The importance of this finding is supported by the fact that MHC class I KO mice are not protected by immunization with *Pb* γ-spz when exposed to a high challenge dose [27]. It has also been shown previously [38] that the activation of CD8^+^ T cells and polarization toward Th1 activity is dependent on IL-12 as it induces T cell expansion and is required for IFN-γ production. In the present study, an essential role for IL-12 in the DC-mediated activation of hepatic CD8^+^ T cells is suggested by the fact that the hepatic cCD8α^+^ DC were the main producers of IL-12. Moreover, anti-IL-12 antibody inhibited both the *in vitro* induced expression of the CD44^hi^CD45RB^lo^ activation phenotype on CD8^+^ T cells and IFN-γ production in co-cultures of CD8^+^ T cells and cCD8α^+^ DC. The additive/synergistic inhibitory activities of the anti-MHC class-I and anti-IL-12 antibodies indicate that both MHC class I:peptide-complexes and IL-12 were required for the activation of the hepatic CD8^+^ T cells by cCD8α^+^ DC. Our observations are firmly supported by many previous findings demonstrating the importance of IL-12 in protection against malaria liver-stage infection via its ability to directly induce IFN-γ production necessary for protection against liver-stage infection [47], [48], [49].

It is now well accepted that protective immunity induced by attenuated Plasmodia sporozoites is multi-factorial involving antibodies, CD4^+^ T cells as well as other lymphocytes, but that the CD8^+^ T cells are the main effectors against pre-erythrocytic infection. An essential question remains, therefore, whether the activation of liver-stage Ag-specific CD8^+^ T cells by DC occurs in the liver or in an immune organ, such as the draining celiac LN, after which the effector CD8^+^ T cells are recruited to the liver. Although our study addressed neither the issue of CD11c^+^NK1.1^−^ DC migration into the liver from other tissues nor their potential differentiation from cells such as CD11c^+^NK1.1^+^ DC that are constitutively present in the livers of naïve mice [50], nonetheless such discussion is warranted in order to reach a better understanding of the individual immune events and their integration into the overall mechanisms that culminate in protective immunity.

According to evidence from studies of immune responses elicited by model protein Ags, viral and microbial Ags as well as from studies of responses to Plasmodia Ags, three different scenarios can be considered: (1) DC acquire and present parasite Ag to CD8^+^ T cells in the draining LN and subsequently the CD8^+^ T cells migrate to the liver; (2) DC acquire parasite Ags in the liver after which they migrate to the LN to activate CD8^+^ T cells and then both cells migrate back to liver; and (3) CD8^+^ T cell:DC interactions occur exclusively in the liver, where DC cross-present liver stage Ag to liver CD8^+^ T cells.

According to the first scenario, immature celiac LN DC with enhanced capacity for Ag uptake, encounter Plasmodia Ags, including those associated with sporozoites that are en route to invade the liver. Among the available Ag for uptake by DC, the CS protein might be the primary candidate as it readily sloughs off but is also quickly replaced on sporozoites. Although uptake of other candidate Plasmodia Ags is quite possible, priming of CD8^+^ T cells specific for *Py* CS protein has recently been observed in the draining LN after vaccination with *Py* γ-spz [16]. Following Ag uptake, the DC mature and activate LN CD8^+^ T cells that then migrate to the liver to perform their effector function during infection. Although our observation of the late emergence of CD44^hi^CD45RB^lo^ CD8^+^ T cells in the liver could be accommodated by this scenario, the incompatibility lies with the Ag specificity as liver CD8^+^ T cells do not recognize peptides from *Pb* CS protein (Krzych U, unpublished data). Other sporozoite or early liver-stage Ag specificities are possible, but they have not been tested.

According to the second scenario, liver resident immature DC take up liver-stage Ag from the apoptotic hepatocytes invaded by *Pb* γ-spz. The Ag-loaded and matured DC then migrate to the liver draining LN, where they engage in the induction of CD8^+^ T cells that subsequently migrate to the liver during infection to perform the effector function. In support of this scenario, influenza-specific CD8+T cells that have been activated in the LN perform optimal effector function only in the presence of Ag-loaded DC in the lung [51]. Although quite attractive, it has been shown that migratory DC do not normally express the CD8α^+^ phenotype [52] and the cCD8α^+^ DC were the major activators of CD8^+^ T cells in our system. In addition, according to the results from a preliminary study, the draining LN contains a very small proportion of activated CD8^+^ T cells following i.d. or i.v. immunizations with *Pb* γ-spz.

The third model proposes that cross presentation of the aborted liver-stage Ags by liver DC and the subsequent activation of liver CD8^+^ T cells occurs uniquely in the liver. The possibility of the local activation of CD8^+^ T cells by liver-stage Ag is quite attractive for several reasons, but especially because it enables a rapid response in the event of parasite invasion of the liver that occurs within 2 mins following i.v. challenge [53]. Furthermore, although activated CD8^+^ T cells home from other regions of the body to the liver where they become eliminated as a way of purging their destructive effector mechanism [54], there is also evidence from other studies showing that direct activation of naïve CD8^+^ T cells can indeed occur in the liver [55], [56], [57]. The accumulation of CD11c^+^NK1.1^−^ DC in the liver could be explained on the basis of an inflammatory response, i.e., production of IL-12 by KC shortly after priming with *Pb* γ-spz [43]. Others have also shown that recruitment of DC to the liver is mediated by soluble factors such as MIP-1α and IL-12, possibly released from KC as DC recruitment is absent in KC cell-depleted animals [58]. KC also trap and maintain DC in the liver via selective adhesion involving N-acetylgalactosamine-mediated interactions [59]. The co-presence of cCD8α^+^ DC and CD8^+^ T cells in the liver and the proximity of liver-stage Ags from the apoptotic *Pb* γ-spz invaded hepatocytes provide all the necessary elements for the initiation of adoptive immune responses in a non-lymphoid organ that is the venue for infection with Plasmodia parasites.

## Materials and Methods

### Mice

Female C57BL/6 mice (6–8 weeks old) were purchased from The Jackson Laboratory (Bar Harbor, ME) and were housed at the Walter Reed Army Institute of Research (WRAIR) animal facility and handled according to institutional guidelines. All procedures were reviewed and approved by the Animal Care and Use Committee of the institute and were performed in a facility accredited by the Association for Assessment and Accreditation of Laboratory Animal Care International.

### Sporozoites, immunizations and parasitemia

*Pb* sporozoites were isolated from mosquitoes as described previously [12] Sporozoites were attenuated by exposure to 15,000 rad of γ-radiation (Cesium-137 source Mark 1 series or Cobalt-60 Model 109; JL Shepard & Associates, San Fernando, CA). As previously described [12], [30], mice were primed i.v. with 75K *Pb*γ−spz followed by two i.v. boost immunizations of 20K *Pb*γ−spz administered 1 week apart. Control mice were primed and boosted with material from sham dissected mosquitoes that represented an equivalent number of infected mosquitoes. For challenge experiments, mice were infected i.v. with a low dose of either 250 or 1000 infectious sporozoites or a high dose of 10, 000 infectious sporozoites approximately one week after the last boost immunization. Thin blood smears were prepared from individual mice starting on day 2 after the challenge and the percentage of parasitized red blood cells was determined microscopically.

### Antibodies

The following mAbs anti-NK1.1 FITC, NK1.1 PE, CD40 FITC, CD80 FITC, CD86 FITC, H-2K^b^ FITC, I-A^b^ FITC, DEC-205 FITC (Serotec, Raleigh, NC.), CD45RB FITC, CD44 PE, B220/CD45RB PE, B220/CD45RB PerCP, CD8α PerCP, CD8α Pacific Orange (Caltag, Burlingame, CA.), CD11c APC, CD3 biotin, CD3e PE, CD3e Pacific Blue (eBioscience, San Diego, CA.), CD8b PE, CD19 biotin, CD19 PE, CD11c biotin, Gr-1 FITC, F4/80 PerCP (Biolegend, San Diego, CA.), F4/80 biotin, Gr-1 biotin, NK1.1 biotin, CD16/CD32 (Fc block), purified anti-IL12p40 (C17.8), purified anti-mouse H-2K^b^/H-2D^b^ (28-8-6) and isotype controls and anti-biotin microbeads (Miltenyi Biotec, Auburn, CA.) were (unless stated otherwise) obtained from (BD Pharmingen, San Diego, CA)

### Isolation of IHMC

Mice were euthanized by CO_2_ inhalation. Livers were exposed and the inferior vena cava was cut for blood outflow. The livers were perfused with 10ml PBS [32], removed and pressed through a 70 µM nylon cell strainer (BD Labware, Franklin Lakes, NJ) and the liver cell suspension was processed as previously described [12], [30]. Briefly, the liver cells were washed 1× in PBS, resuspended in PBS containing 35% Percoll (Amersham Pharmacia Biotec, Uppsala, Sweden) and centrifuged at 2,000 rpm for 20 min. Erythrocytes in the cell pellet were lysed with RBC lysis buffer (Sigma, St. Louis, MO) and the remaining hepatic mononuclear cells (HMNC) were washed and resuspended in complete RPMI 1640 medium.

### Isolation of hepatic CD11c^+^NK1.1^−^ DC

CD11c^+^NK1.1^−^ cells were isolated as previously described [2]. Briefly, HMNC were incubated with a cocktail of biotinylated (anti-CD3e, CD19, NK1.1, Gr-1, F4/80) mAb for 15 minutes at 4°C. Following washing in buffer (PBS containing 2mM EDTA and 0.5% BSA), the cells were magnetically labeled with anti-biotin microbeads for 15 minutes at 4°C. The CD11c^+^ cells were enriched by negative isolation on a MS column (Miltenyi Biotec) according to the manufacturer's instructions. The purity and viability of the enriched CD11c^+^NK1.1^−^ preparation were assessed by flow cytometry and trypan blue exclusion,.and were found to be ∼94% and >96% respectively.

### Isolation of CD11c^+^NK1.1^−^ DC subpopulations

For isolation of cCD8α^+^ DC or pDC, enriched hepatic CD11c^+^NK1.1^−^ cells were labeled with CD8α (Ly-2) microbeads or CD45R (B220) microbeads and the labeled cells isolated by positive selection on MS columns. For isolation of cCD8α^−^DC, CD11c^+^NK1.1^−^ cells were labeled with a cocktail of CD8α (Ly-2) and CD45R (B220) microbeads and isolated by negative selection on MS columns. The purity of the DC subpopulations was >95% and viability was >97%.

### RT-PCR

Total RNA was extracted from purified splenic CD8^+^T cells and purified liver CD8α^+^CD11c^+^ cells using an RNeasy Plus Mini Kit (Qiagen, Valencia, CA) and treated with DNase. RNA concentration and quality control were determined by UV spectrophotometry and denaturing agarose gel electrophoresis. For all samples, cDNA was synthesized from total RNA using High Capacity cDNA Reverse Transcription Kit (Applied Biosystems, Foster City, CA) according to the manufacturer's instructions. The qPCR for CD8α chain mRNA and CD8β chain mRNA was performed using SuperArray's RT^2^ Real-Time SYBR Green/Rox PCR master kit, CD8α (PPM04031A), CD8b1 (PPM04032E) primer mixes, and Applied Biosystems 7500 Fast Real-Time PCR System. The temperature profile was 10 min at 95°C followed by 40 cycles of (95°C, 15 sec; 60°C, 60 sec). Melting curve analysis was performed after qPCR for quality control purposes.

### Flow cytometry

For evaluation of T cell activation, four-color staining of HMNC and splenic MNC was performed using a combination of the following mAb: FITC-anti-CD45RB (16A), PE-anti-CD3 (17A2), PerCP-anti-CD8α (53-6.7), and APC-anti-CD44 (IM7). For identification of CD11c^+^NK1.1^−^ DC; HMNC and splenic MNC were stained using a combination of the following mAb: FITC-anti-NK1.1 (PK136), PE-anti-CD3e (17A2), PE-anti-CD8b (53-5.8), PerCP-anti-CD45R/B220 (RA3-6B2), Pacific Orange-anti-CD8α, and APC-anti-CD11c (HL3). For evaluation of DEC-205, MHC and costimulatory molecules, HMNC were analyzed using a combination of the following mAb: FITC-anti-DEC-205 (NLDC-145), FITC-anti-CD40, FITC-anti-CD80 (16-10A1), FITC-anti-CD86 (GL-2), FITC-anti-IA^b^ (AF6-120.1), FITC-anti H-2K^b^, PE-anti-CD3e, PE-anti-CD8b, PerCP-anti-CD45R/B220, Pacific Orange-anti-CD8a, and APC-anti-CD11c. For evaluation of the purity of the CD11c^+^CD8α^+^ cells, the positively isolated cells were stained using a combination of the following mAb: FITC-anti-NK1.1, PE-anti-CD11c, PerCP-anti-CD19, Pacific Blue-anti-CD3e and Pacific Orange-anti-CD8α. Briefly, 2–10×10^5^ cells were resuspended in cold assay buffer (PBS containing 1% BSA (Sigma) and 0.01% sodium azide) and incubated with anti-FcR 24G2 and 0.5 µg of the relevant mAb at 4°C for 30 min. Cells were washed and resuspended in cold assay buffer. Flow cytometry analyses of T cells and of DC were performed on a FACSCalibur (BD Biosciences) and an LSRII (BD Biosciences) flow cytometer, respectively. Data analysis was performed using FlowJo 8.1.0 software (Tree Star, Inc., Ashland, OR).

### *In vitro* cultures

CD11c^+^NK1.1^−^ DC (5×10^5^) or purified DC subpopulations (5×10^5^) from livers or spleens of *Pbγ-*spz-immunized mice were co-cultured with naïve hepatic or splenic CD8^+^ T cells (1×10^6^) in a 1ml volume of complete culture media (RPMI 1640 supplemented with 10% heat-inactivated FCS, 10mM Hepes, 100U of penicillin, 100 µg of streptomycin and 4mM L-glutamine) in a 48-well tissue culture plate for 4 days. A ratio of 1∶2 of hepatic DC:CD8^+^ T cells yielded the highest percentage of recovered viable CD8^+^ T cells and was used in all experiments. For blocking experiments, antibodies against lL-12p40 (clone C17.8) and H-2K^b^/H-2D^b^ (clone 28-8-6) were used at 5 µg/ml. Cells and culture supernatants were harvested at day 4 for determination of CD8^+^ T cell activation and cytokine production, respectively.

### CFSE labeling

Purified splenic CD8^+^ T cells were incubated with 2 µM CFSE (Molecular Probes – Invitrogen, Eugene, OR.) for 7 min at room temperature. Cells were washed three times with 10ml complete culture medium. After the final wash, the cells were resuspended in complete culture medium and viability was >98%. 1×10^6^ CFSE-labeled splenic CD8+T cells were co-cultured with 5×10^5^ purified cCD8α^+^DC, cCD8α^−^DC and pDC isolated from livers of *Pbγ-*spz-immunized-challenged mice in a 1ml volume of complete culture media in a 48-well tissue culture plate for 4 days. Cells were harvested, washed and stained with PE-anti-CD45RB (16A), PerCP-anti-CD3 (17A2), APC-anti-CD44 (IM7) and Pacific Orange-anti-CD8a for evaluation of T cell activation.

### Morphology

Freshly purified hepatic cCD8α^+^DC, cCD8α^−^DC and pDC were cytospun onto glass slides (1 min at 1000rpm) and fixed in methanol for 15 min at room temperature. Slides were stained with Giemsa (Sigma Diagnostics, St Louis, MO) for 20 min and photographed.

### IL-10 and IL-12p40 determination

The presence of IL-10 and IL-12p40 were determined using IL-10 and IL-12p40 ELISA kits (BD Biosciences, San Diego, CA) according to the manufacturer's instructions.

### IFN-γ Determination

IFN-γ was determined using an R&D ELISA kit **(**R&D Systems, Minneapolis, MN**)** and according to the manufacturer's instructions.

### Adoptive transfer

To evaluate the protective effect of hepatic DC against liver-stage infection, purified CD11c^+^NK1.1^−^ DC (1×10^6^) from livers of *Pb* γ−spz-immunized mice were transferred (i.v.) into naïve C57BL/6 mice. Seven days after the adoptive transfer, recipient mice were challenged with 250 or 1000 infectious sporozoites. Parasitemia and survival were evaluated from day 2 post-challenge.

### Statistical Analysis

Data are presented as the means±SD, and the differences among groups were analyzed by the Mann-Whitney *U* test using the Graphpad Prism software. A value of p<0.05 was considered significant.
